# Contributions of the Complementarity Determining Regions to the Thermal Stability of a Single-Domain Antibody

**DOI:** 10.1371/journal.pone.0077678

**Published:** 2013-10-15

**Authors:** Dan Zabetakis, George P. Anderson, Nikhil Bayya, Ellen R. Goldman

**Affiliations:** 1 Center for Bio/Molecular Science and Engineering, US Naval Research Laboratory, Washington, DC, United States of America; 2 Science and Engineering Apprenticeship Program, American Society for Engineering Education, Washington, DC, United States of America; Russian Academy of Sciences, Institute for Biological Instrumentation, Russian Federation

## Abstract

Single domain antibodies (sdAbs) are the recombinantly-expressed variable domain from camelid (or shark) heavy chain only antibodies and provide rugged recognition elements. Many sdAbs possess excellent affinity and specificity; most refold and are able to bind antigen after thermal denaturation. The sdAb A3, specific for the toxin Staphylococcal enterotoxin B (SEB), shows both sub-nanomolar affinity for its cognate antigen (0.14 nM) and an unusually high melting point of 85°C. Understanding the source of sdAb A3’s high melting temperature could provide a route for engineering improved melting temperatures into other sdAbs. The goal of this work was to determine how much of sdAb A3’s stability is derived from its complementarity determining regions (CDRs) versus its framework. Towards answering this question we constructed a series of CDR swap mutants in which the CDRs from unrelated sdAbs were integrated into A3’s framework and where A3’s CDRs were integrated into the framework of the other sdAbs. All three CDRs from A3 were moved to the frameworks of sdAb D1 (a ricin binder that melts at 50°C) and the anti-ricin sdAb C8 (melting point of 60°C). Similarly, the CDRs from sdAb D1 and sdAb C8 were moved to the sdAb A3 framework. In addition individual CDRs of sdAb A3 and sdAb D1 were swapped. Melting temperature and binding ability were assessed for each of the CDR-exchange mutants. This work showed that CDR2 plays a critical role in sdAb A3’s binding and stability. Overall, results from the CDR swaps indicate CDR interactions play a major role in the protein stability.

## Introduction

Single-domain antibodies (sdAbs) are small recombinantly-produced binding elements derived from the heavy-chain-only antibodies produced by camelids and sharks [1–4]. Composed of an individual variable binding domain of about 110-120 amino acids these fully functional antibody fragments are capable of production by bacterial expressions systems and, since they lack quaternary structure, are capable of refolding after thermal denaturation [5–9]. In addition, certain sdAbs exhibit high thermal stability, as exemplified by the previously described sdAb A3 with a melting temperature of 85°C [10].

**Figure 1:**
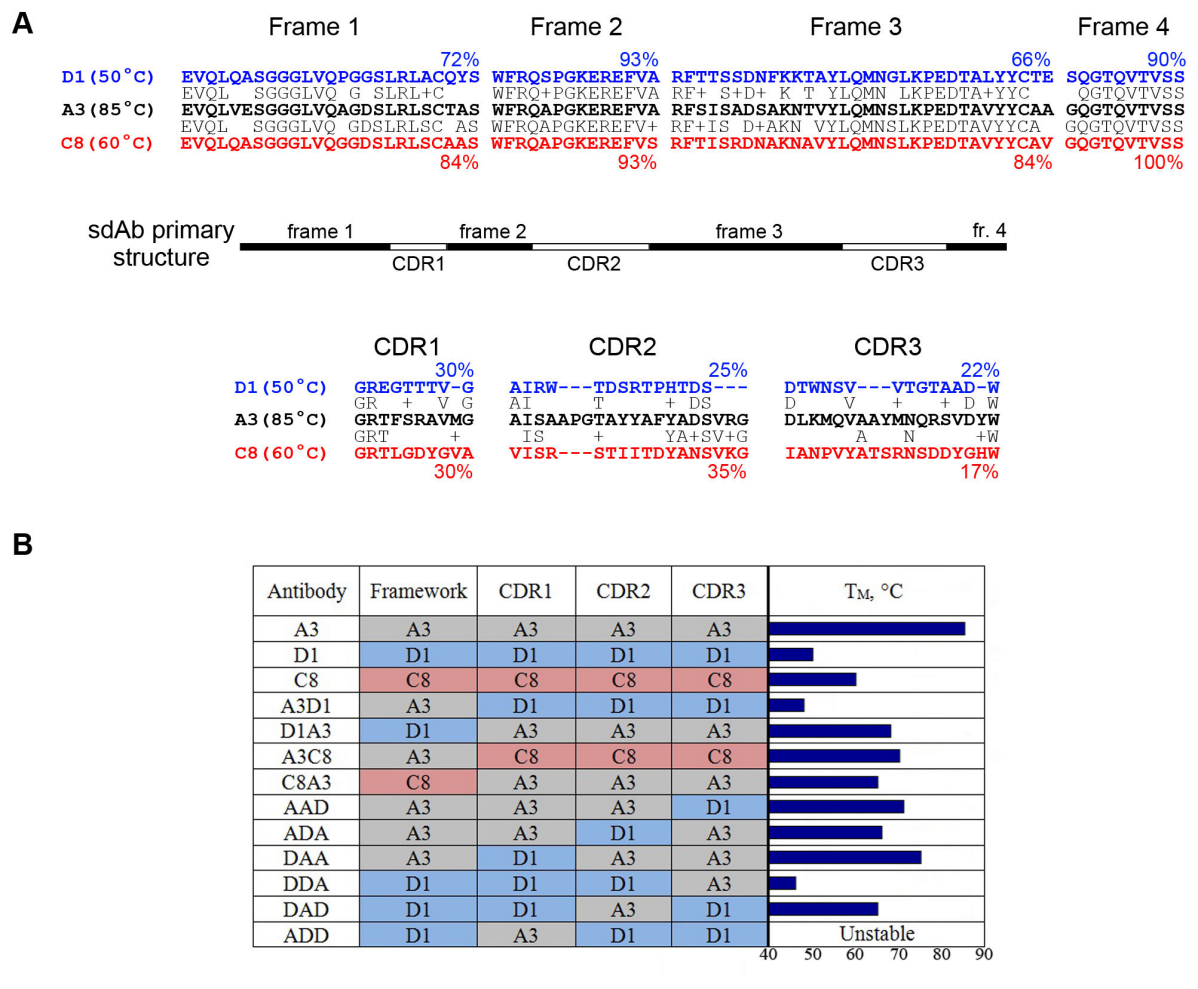
Primary structure and sequence of sdAbs used in this study. A) The overall primary structure of sdAbs is shown schematically with alternating framework and CDRs. Melting temperature for the wildtype sdAbs is given in parentheses next to the name. The framework regions are grouped together above the schematic while the CDRs are shown below. The percent identity of sdAb D1 and sdAb C8 toward sdAb A3 is shown for each region. B) Construct identifications are shown schematically for all hybrid antibodies in this study. Regions are color coded for clarity. Observed melting point is shown as a bar graph. Detailed measurements are presented in Table 1.

SdAb A3 was selected from a library of phage-displayed sdAbs derived from an immunized llama and shows high affinity and specificity for Staphylococcal enterotoxin B (SEB) [10,11]. The sequence of this sdAb is shown in Figure 1 and reveals a typical structure for V_H_H, variable domains derived from heavy-chain-only antibodies of camelids. As in conventional variable heavy domains, there are four highly-conserved framework regions alternating with highly-variable complementarity determining regions (CDRs) which embody the specific binding interaction of the antigen-antibody complex. In this study we compare sdAb A3 to both low-melting sdAb D1 (50°C) and moderate-melting sdAb C8 (60°C), whose sequences are also shown in Figure 1 for comparison. Both sdAb D1 and sdAb C8 have binding specificity for ricin which can be used to distinguish functional activity from sdAb A3 [12–14]. 

**Table 1:** Melting temperature and binding affinity.

Antibody	Framework	CDR1	CDR2	CDR3	T_M_, °C	SEB Affinity, M	Ricin Affinity, M
A3	A3	A3	A3	A3	85	1.4 x 10^-10^	--
D1	D1	D1	D1	D1	50	--	5.0 x 10^-10^
C8	C8	C8	C8	C8	60	--	2.0 x 10^-11^
A3D1	A3	D1	D1	D1	48	--	9.4 x 10^-9^
D1A3	D1	A3	A3	A3	68	5.6 x 10^-10^	--
A3C8	A3	C8	C8	C8	70	--	7.2 x 10^-10^
C8A3	C8	A3	A3	A3	65	2.7 x 10^-9^	--
AAD	A3	A3	A3	D1	71	1.2 x 10^-8^	--
ADA	A3	A3	D1	A3	66	--	--
DAA	A3	D1	A3	A3	75	1.5 x 10^-9^	--
DDA	D1	D1	D1	A3	46	--	--
DAD	D1	D1	A3	D1	65	2.0 x 10^-8^	--
ADD	D1	A3	D1	D1	Unstable	--	--

Binding not observed.

The features of a sdAb provide a favorable opportunity to investigate the relationship between functional activity and structural stability. The alternating conserved and variable regions allows for swapping of sequences with high confidence that the resulting hybrid will retain the overall secondary structure and possibly also the binding functionality. To this end the CDRs can be exchanged (as a group or individually) between sdAbs of differing affinity and melting temperature in order to analyze these relationships.

Swapping CDRs (also called CDR grafting) is a technique that has been utilized for the humanization of murine antibodies [15], and also for the construction of more stable conventional antibody fragments [16,17]. In addition, it has been applied to sdAbs in an effort to identify a universal sdAb scaffold for improved stability [18], as well as humanization of camelid sdAbs [19]. 

Between sdAb A3 and sdAb D1 there is a 35°C difference in melting point. Two alternate hypotheses are immediately suggestive as explanations for this difference. First, since the framework regions are highly conserved one might predict that small differences in these sequences could have a dramatic effect on stability. The sequence identity between the framework regions of sdAb A3 and sdAb D1 varies from 66% to 93%, and it may be here that we would expect to find the source of melting temperature variability.

On the other hand, it may be felt that due to the high conservation of framework regions there has already been an evolutionary rejection of unstable sequences. And since the CDRs are both highly variable and selected for binding affinity rather than stability it may be proposed that variation in melting point between sdAbs is due to these regions.

In this work we constructed a series of CDR-swap mutants with the goal of understanding the contribution of the framework and CDRs to the stability of sdAb A3. Through this work we were able to achieve insights into the role of these regions in sdAb stability and affinity.

## Materials and Methods

### Reagents

Staphylococcal enterotoxin B (SEB) was purchased from Toxin Technologies and ricin was purchased from Vector Laboratories. Cloning enzymes were from New England Biolabs. Reagents for surface plasmon resonance were purchased from Bio-Rad. All other chemicals were purchased from Sigma-Aldrich. Wildtype antibodies were derived from V_H_H phage-display libraries prepared from the peripheral blood lymphocytes of immunized llamas maintained by Triple J Farms and have been described previously [10,12].

### DNA Synthesis and Sequencing

Antibody expression vectors were constructed in pET22b+ (EMD Millipore) and based on the sequences given in Figure 1. DNA sequences are in GenBank, accession numbers KF553633, KF553634 , and KF553635 corresponding to A3, C8, and D1 respectively. For each antibody the sequence was synthesized (MWG Eurofins Operon) and cloned from pCR2.1 into pET22b+ by way of an NcoI/NotI fragment. The correct sequence was verified by sequencing (MWG Eurofins Operon).

### Protein Expression

Protein was produced by expression in *E. coli* strain Rosetta(DE3) from EMD. Expression vector pET22b+ directs proteins to the periplasmic space. An osmotic shock protocol was performed and protein purified by immobilized metal ion affinity chromatography followed by size exclusion chromatography as described previously [10,13]. Typical yields for sdAb A3, sdAb C8, and sdAb D1 were between 4 and 11 mg of purified protein from 500 ml of bacterial culture; protein yields of the CDR-swap mutants were generally within this same range.

### Melting Point Determination

The melting points of all antibodies were measured by circular dichroism (CD) using a Jasco J-815 CD spectropolarimeter equipped with a PTC-423S single position peltier temperature control system as described previously [10,13]. Briefly, samples (~12 µg/mL) were prepared by dilution or dialysis versus de-ionized water. Melting point data were acquired at a single wavelength between 200 and 205 nm, at a temperature rate of 2.5°C/min over the range of 25°C to 95°C. Data as reported is accurate to within 1°C, and primary data for all measurements are included in Figure S1.

### Antibody Binding Affinity

Surface plasmon resonance (SPR) kinetic measurements were performed using the ProteON XPR36 (Bio-Rad). For testing the kinetics of the antibody constructs, a GLC chip was coated with SEB on two lanes and ricin on two lanes, and the antibodies tested essentially as previously described [11,20,21], except that due to the high affinity of sdAb A3 it was necessary to regenerate the surface between runs using a 36 second exposure to10 mM glycine HCl pH 2.5 with 0.05% SDS. SEB was resistant to these conditions, however ricin was not, so binding to ricin was evaluated first with the surface being regenerated using 10 mM glycine HCl pH 2.5. Data for all measurements are included in Figure S2; the error on the fit is typically less than 10% which is less than the variance between replicate samples which typically agreed within a factor of 2.

## Results and Discussion

### The CDRs determine the melting point difference between sdAb A3 and sdAb D1

SdAb A3 is unusually stable, with a melting point of 85°C (see reference [10] Figure 7) and has affinity to SEB. In order to elucidate the particular regions and sequences responsible for this stability, hybrid proteins were constructed using sdAb D1 sequences as an alternative. SdAb D1 has specificity for ricin and an unusually low melting temperature of 50°C. Therefore, by swapping the CDR and framework regions of these antibodies we are able to determine the relative contribution to overall thermal stability.

**Figure 2:**
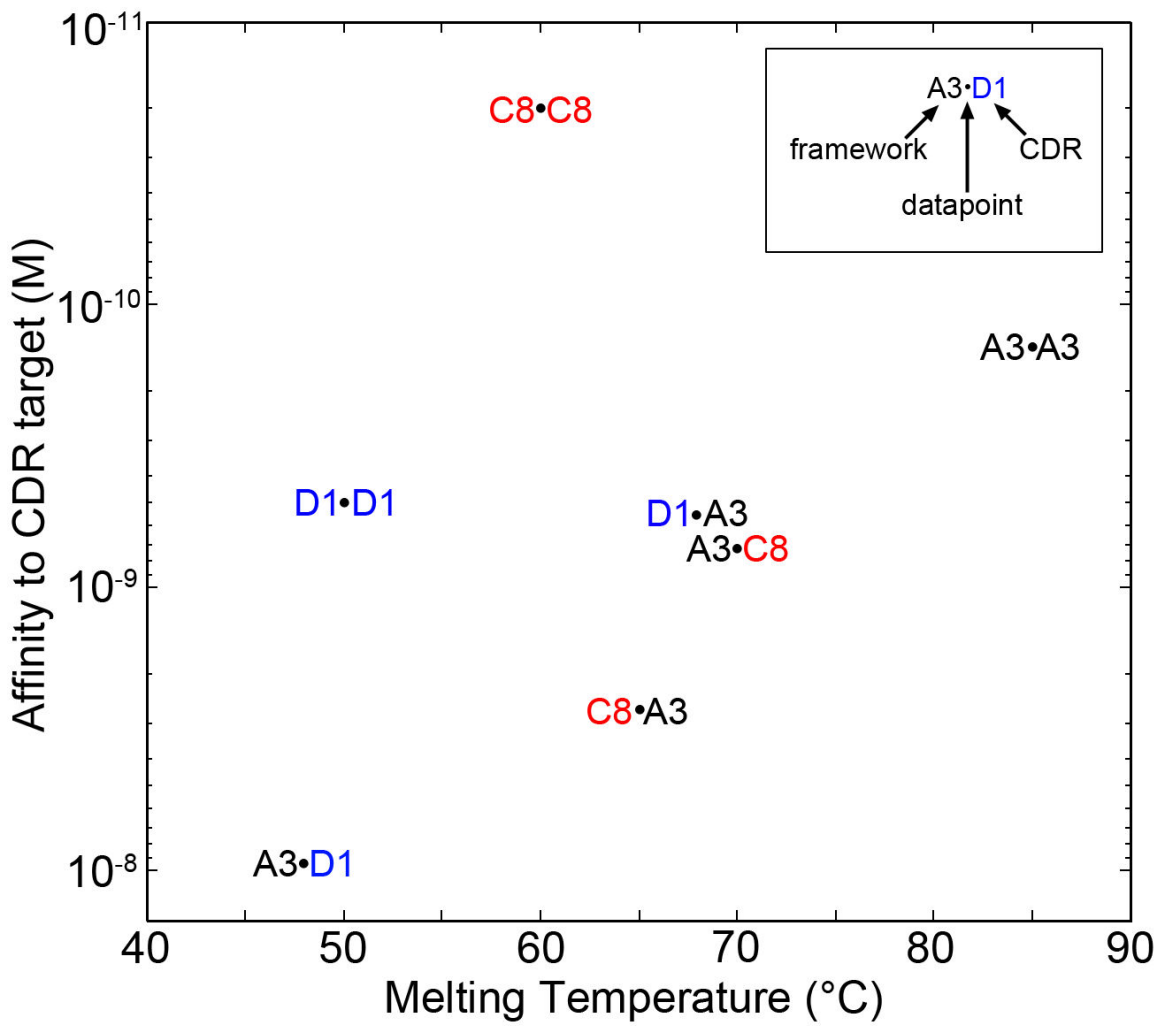
CDR swaps lower affinity but may increase or decrease melting temperature. Melting temperature for sdAbs A3, D1 and C8, as well as full CDR swaps, are plotted against affinity. For each data point the framework origin is indicated before the dot while the CDR origin is indicated after the dot. The affinity shown is that towards the target specified for the CDR origin antibody.

Hybrid construct A3D1 consists of the framework regions from sdAb A3 and the CDRs from sdAb D1 and was expressed in *E. coli* from vector pET22b+. Likewise construct D1A3, consisting of the framework of sdAb D1 and the CDRs of sdAb A3, was synthesized and expressed. The results are shown in Figures 1 and 2 and in Table 1. While both hybrids melted lower than sdAb A3, A3D1 melted 37°C lower while D1A3 melted only 17°C lower. Compared to the low melting temperature of sdAb D1, the change in melting point for the hybrids was -2°C and +18°C, respectively. As expected, the binding specificity follows the CDR regions [18,22], although with a significant reduction in affinity.

Since replacing the CDRs of sdAb A3 with sequences from sdAb D1 results in a dramatic lowering of melting point, while replacing the framework sequences with those from sdAb D1 results only in a lesser reduction, we conclude that it is the CDR sequences that contribute the most to the unusually high thermal stability of sdAb A3. Additional factors are no doubt also involved, such as cooperative folding between the framework regions and the CDRs. Because the CDRs and framework are not completely independent units, swapping of the CRDs may influence folding and stability of the remote framework regions, but would be difficult to detect by this method. 

### CDR2 is the major contributor to the stability of sdAb A3

Since the three CDRs are clearly identifiable and separate, it is possible to further dissect the antibody sequence. Six constructs were synthesized and expressed in *E. coli* using the pET22b+ vector and are shown in Figure 1. One CDR was individually introduced from sdAb A3 or sdAb D1 into the other antibody sequence and the hybrid name is given showing the identity of each CDR. The framework is not made explicit in these names but is always the framework of the accepting sequence with two of the three CDRs (i.e. 'AAD' consists of the framework, CDR1 and CDR2 from sdAb A3, and CDR3 from sdAb D1).

Among these six constructs only hybrid ADD was problematical. Production of this protein was difficult and yield was low (<1mg/500ml culture). In addition, the CD spectrum was aberrant compared to other sdAbs and no clear melting inflection point was observed (CD spectrum and melting data are shown in Figures S3 and S1 respectively). From this we conclude that ADD is unstable in bacterial production and takes on an improperly folded structure.

Evaluating the melting temperatures for the remaining five constructs indicates that CDR2 is the most important region contributing to the thermal stability of sdAb A3. Swapping one CDR from sdAb D1 into the sdAb A3 sequence showed the most effect on melting point when the swapped region was CDR2 (a lowering of the melting point by 19°C), although in all cases the effect was significant. The results of swapping a single CDR from sdAb A3 into sdAb D1were more clear. Introducing CDR1 resulted in an unstable, improperly folded antibody. Swapping in CDR3 also destabilized the antibody, reducing the melting point 4°C. Yet replacing the sdAb D1 CDR2 with the sequence from sdAb A3 resulted in a 15°C increase in melting temperature.

From this data we conclude that CDR2 is the most important region of sdAb A3 contributing to thermal stability, and that the CDRs from sdAb D1 are not individually destabilizing (since none of them resulted in a low melting point when introduced into sdAb A3). 

### Thermal stability cannot be freely engineered without regard for affinity

The binding target and affinity of the six hybrid constructs was determined by surface plasmon resonance kinetic measurements. No affinity to ricin was observed for any of the antibodies, suggesting that the binding site of sdAb D1 is distributed among all the CDRs or else is highly sensitive to small changes in structure. 

For SEB, specific binding was observed to follow the presence of CDR2. Hybrid DAD, consisting of only CDR2 of sdAb A3 within the sequence of sdAb D1 showed affinity to SEB and not ricin, although at a magnitude 140-fold lower than wildtype sdAb A3. CDR1 is least important, when replaced by sdAb D1 sequence in hybrid DAA the result was the smallest reduction in affinity (a 11-fold reduction with respect to the wildtype sequence). CDR3 is of intermediate importance for binding SEB. Alone or in combination with CDR1, CDR3 could not confer binding. When sdAb D1 sequence was used to replace the CDR3 of sdAb A3 there was an 86-fold reduction in affinity.

These results show that both the binding specificity and the unusual thermal stability reside primarily in CDR2. This suggests that the thermal stability was a fortuitous result of selection for the appropriate antibody in the llama immune response. Unfortunately it also indicates that thermal stability of the type achieved by sdAb A3 cannot be independently introduced into other antibodies and that stability cannot be freely engineered without possibly disrupting the binding function of the protein.

Our finding that CDR2 is most important for the affinity of sdAb A3 is in contrast with several sdAbs targeting hen egg lysozyme in which CDR3 is critical for antigen binding. The CDR3regions of these sdAbs have been shown to enable binding in the absence of the other CDRs when moved to a non-target binding sdAb or even a non-variable domain scaffold [23,24].

### The effect of CDR swapping on a moderate melting point antibody is limited

SdAb C8 is an anti-ricin antibody of moderate melting temperature (60°C). Since most sdAbs have melting temperatures in the 60°-70°C range it is an important question whether swapping regions with a thermally-stable antibody will have the same effects as exchanges between sdAbs possessing high and low thermal stability. In the simplest model one would assume that the effects should be the same. If swapping CDRs from sdAb A3 into the framework of sdAb D1 results in an 18°C increase in melting temperature then placing the CDRs into the framework of sdAb C8 should result in an antibody melting at 78°C.

On the other hand it may be argued that antibodies which are not inherently unstable will not benefit from region swapping since it may be true that every beneficial change is opposed by a simultaneous detrimental change during any sequence alteration. We might therefore expect that for antibodies with moderate melting points the effect of any swap will be less than for antibodies of extreme (high or low) melting points.

In order to test these hypotheses, hybrid antibodies were synthesized swapping the CDR and framework regions of sdAb A3 and sdAb C8. The results are plotted on Figure 2. In both cases the affinity to the target specified by the CDRs is reduced about 20-fold. However, both melting temperatures remained within the moderate range. In contrast to the results observed for swaps with sdAb D1, the hybrid C8A3 had a lower melting temperate than A3C8 (i.e. the sdAb A3 CDRs had a smaller effect than the framework). The difference was only 5°C and both hybrid melting points are higher than sdAb C8, yet still within the moderate range.

We conclude from this result that the second hypothesis is true and that when starting with reasonably stable antibodies the balance between beneficial and detrimental alterations results in melting behavior changes of a lower magnitude than might be expected and furthermore are difficult to predict.

### Extremes of melting point variability may be due to specific sequences while moderating effects may be diffuse

The conclusions drawn from this study as a whole illustrate several features of sdAb stability and function that may be general. First, this work confirmed that CDR swapping is a viable method for attempting to increase the thermal stability of an antibody. In all cases the CDRs continued to bind their expected targets, albeit at a reduced affinity. In the case of sdAb C8 the thermal stability was increased 10°C by replacing the framework with that of sdAb A3. The same swap with sdAb D1 resulted in a drop in stability. Therefore it seems true that while CDR swapping will work in some cases there is significant and, at this time, unpredictable variability.

Also, we showed that the most important region conferring stability to sdAb A3 was CDR2 and that this region was also an important determinant of specificity. If it is generally true that variability in melting temperature is the result of selection for affinity to specific antigens then stability will not be subject to independent engineering. We observed that swapping the framework of sdAb A3 into sdAb D1 did not raise the melting point. Swapping in the CDRs did, but that swap also changed the target specificity as expected. In fact, no construct using only one or two of the sdAb D1 CDRs was found to bind the target. So, while this process was successful in identifying the regions critical for protein stability, we were unable to engineer a variant of sdAb D1 with increased stability while retaining wildtype specificity.

Considering the contrast between antibodies with extreme melting points (sdAbs A3 and D1) and one with a moderate temperature (sdAb C8) there appears to be a distinction between specific and diffuse effects. Either high or low melting behavior may be caused by one or more particular interactions that confer particular stability or instability to a protein. Moderate, typical melting points would then be the result of the absence of these extreme interactions with the overall stability dependent on a multitude of average interactions. In this case introduction of a stabilizing interaction might often be offset by the simultaneous induction of destabilizing forces.

Attempts to determine the crystal structure of sdAb A3 are underway and it is expected that the CDR regions will be found most conducive to protein stability. We will then be able to examine the interactions between framework and CDRs and elucidate those residues which contribute most substantially to the overall stability.

## Supporting Information

Figure S1 **Circular dichroism measurements of melting temperature of antibody constructs presented in this paper.** Heating and cooling curves are shown for each mutant.
(DOC)

Figure S2 **Surface plasmon resonance measurements for affinity of the constructs presented in this paper.** The target was SEB for all antibodies except A3D1 and A3C8, for which the target was ricin.
(DOC)

Figure S3 **Circular dichroism spectrum of sdAb ADD showing an atypically flat curve.** The spectrum of sdAb DAA is shown for comparison and reveals a typical sdAb spectrum with positive and negative CD regions between 210 nm and 230 nm, and a large positive peak below 210 nm.
(DOC)
