# Supplementary material for: Contributions of the Complementarity Determining Regions to the Thermal Stability of a Single-Domain Antibody
Source: PLoS One. 2013 Oct 15;8(10):e77678. doi: 10.1371/journal.pone.0077678 (PMC3797041; doi:10.1371/journal.pone.0077678)

Supporting Information

Contributions of the Complementarity Determining Regions to the Thermal Stability of a Single-Domain Antibody

Dan Zabetakis^1^, George P. Anderson^1^, Nikhil Bayya^2^, Ellen R. Goldman^1^*

^1^Center for Bio/Molecular Science and Engineering, US Naval Research Laboratory, Washington, DC

^2^Science and Engineering Apprenticeship Program, American Society for Engineering Education, Washington, DC

Figure S1

Circular Dichroism measurements of melting temperature of antibody constructs presented in this paper. Heating (red) and cooling (blue) curves are shown for each antibody as explained in the Materials and Methods section.

Figure S1.


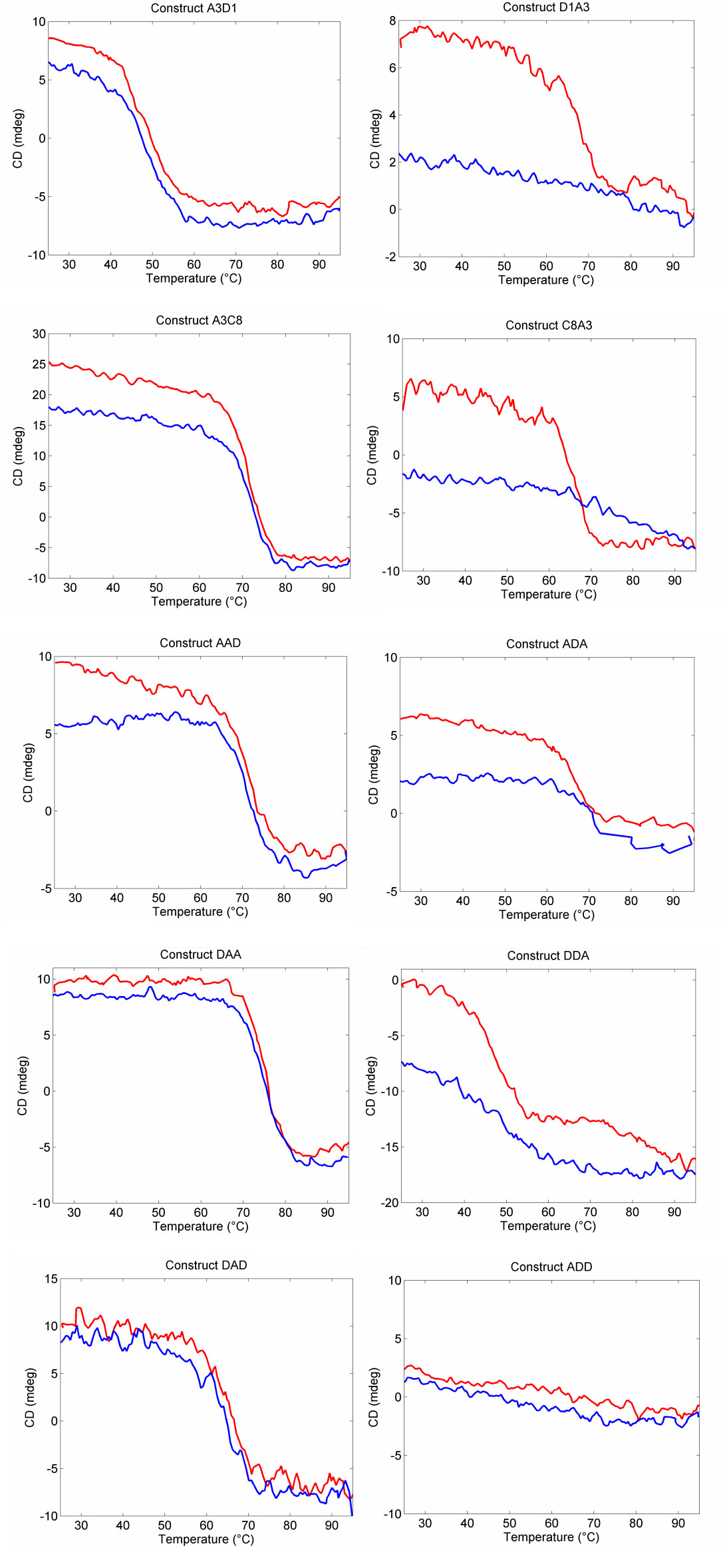

Supplement: Figure S1 — Circular dichroism measurements of melting temperature of antibody constructs presented in this paper. Heating and cooling curves are shown for each mutant. (DOC) [file pone.0077678.s001.doc]
