# Supplementary material for: Contributions of the Complementarity Determining Regions to the Thermal Stability of a Single-Domain Antibody
Source: PLoS One. 2013 Oct 15;8(10):e77678. doi: 10.1371/journal.pone.0077678 (PMC3797041; doi:10.1371/journal.pone.0077678)
Supplement: Figure S2 — Surface plasmon resonance measurements for affinity of the constructs presented in this paper. The target was SEB for all antibodies except A3D1 and A3C8, for which the target was ricin. (DOC) [file pone.0077678.s002.doc]

Supporting Information

Contributions of the Complementarity Determining Regions to the Thermal Stability of a Single-Domain Antibody

Dan Zabetakis^1^, George P. Anderson^1^, Nikhil Bayya^2^, Ellen R. Goldman^1^*

^1^Center for Bio/Molecular Science and Engineering, US Naval Research Laboratory, Washington, DC

^2^Science and Engineering Apprenticeship Program, American Society for Engineering Education, Washington, DC

Figure S2

Surface Plasmon Resonance measurements for affinity of the antibodies presented in this paper. Antibody binding kinetics are shown for different concentrations of each antibody as shown and curve fits and calculations of the K_D_ were performed by the instrument software. Details are in the Materials and Methods section. The target was SEB for all antibodies except A3D1 and A3C8, for which the target was ricin.

Figure S2.
