# Supplementary material for: Contributions of the Complementarity Determining Regions to the Thermal Stability of a Single-Domain Antibody
Source: PLoS One. 2013 Oct 15;8(10):e77678. doi: 10.1371/journal.pone.0077678 (PMC3797041; doi:10.1371/journal.pone.0077678)

Supporting Information

Contributions of the Complementarity Determining Regions to the Thermal Stability of a Single-Domain Antibody

Dan Zabetakis^1^, George P. Anderson^1^, Nikhil Bayya^2^, Ellen R. Goldman^1^*

^1^Center for Bio/Molecular Science and Engineering, US Naval Research Laboratory, Washington, DC

^2^Science and Engineering Apprenticeship Program, American Society for Engineering Education, Washington, DC

Figure S3

Circular Dichroism spectrum of antibody ADD showing an atypically flat curve. The spectrum of antibody DAA is shown for comparison and reveals a typical sdAb spectrum with positive and negative CD regions between 210 nm and 230 nm, and a large positive peak below 210 nm.

Figure S3.


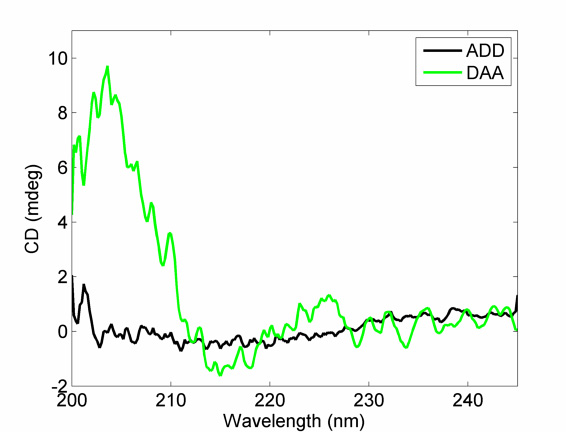

Supplement: Figure S3 — Circular dichroism spectrum of sdAb ADD showing an atypically flat curve. The spectrum of sdAb DAA is shown for comparison and reveals a typical sdAb spectrum with positive and negative CD regions between 210 nm and 230 nm, and a large positive peak below 210 nm. (DOC) [file pone.0077678.s003.doc]
